# External application of mirabilite before surgery can reduce the inflammatory response and accelerate recovery in mild acute biliary pancreatitis

**DOI:** 10.1186/s12876-023-02901-5

**Published:** 2023-08-02

**Authors:** Hao Cai, Jian Du, Cheng Luo, Shengwei Li

**Affiliations:** 1 Department of Hepatobiliary Surgery, The Second Affiliated Hospital of Chongqing Medical University, Chongqing, 400010 China; 2 Department of Hepatobiliary Surgery, Suining Hospital of Traditional Chinese Medicine, Suining city, Sichuan Province 629000 China

**Keywords:** Biliary pancreatitis, Laparoscopic cholecystectomy, Mirabilite, Inflammatory response, Adhesion

## Abstract

**Objective:**

Mild acute biliary pancreatitis (MABP) is one of the most common diseases that require surgical treatment. Previous studies have focused on the timing of laparoscopic cholecystectomy (LC) for MABP. However, the impact of its inflammatory response process on the clinical outcome has been rarely reported. This study aimed to investigate the effect of preoperative external application of mirabilite on the inflammatory response and clinical efficacy in MABP.

**Methods:**

Medical records of patients undergoing LC due to MABP from November 2017 to June 2022 were retrospectively reviewed. Prior to surgery, the control group received the same baseline treatment measures as the study group. The difference was the addition of external application of mirabilite in the study group.

**Results:**

A total of 75 patients were included in the final analysis: 38 patients in the mirabilite group and 37 patients in the control group. Repeated-measures ANOVA (*P* < 0.01) showed that the white blood cell count (WBC) on the 3rd day of admission and the WBC and C-reactive protein (CRP) level on the 5th day of admission decreased rapidly and significantly in the mirabilite group, compared with the control group. The mirabilite group had earlier anal exhaust time. The number of patients in the mirabilite group and control group with gallbladder wall ≥ 3 mm before the operation was 16 (42.11%) vs. 24 (64.86%), *p* = 0.048, respectively; and the number of cases with surgical drain placement was 2 (5.26%) vs. 9 (24.32%), *p* = 0.020, respectively. The intraoperative modified American Fertility Society (mAFS) score of adhesions was lower in the mirabilite group (1.08 ± 0.59 points) than in the control group (1.92 ± 0.60 points), *p* = 0.000. The mirabilite group, compared to the control group, *p* = 0.000, had a short waiting time for surgery (5.68 ± 0.70 days vs. 6.54 ± 0.59 days), short operation time (38.03 ± 5.90 min vs. 48.51 ± 8.37 min), and reduced hospitalization time (8.95 ± 0.96 days vs. 9.84 ± 1.07 days).

**Conclusion:**

This study demonstrated that preoperative external application of mirabilite can reduce the inflammatory response, decrease the edema and peribiliary adhesions at the surgical site, and accelerate recovery in MABP.

## Introduction

 Acute pancreatitis (AP) is a common digestive disease worldwide [1]. The total incidence of AP has increased at an annual rate of 3.07%, resulting in a higher burden on the health care system [2]. AP, which is the main cause of biliary tract disease, is increasing at a rate of 3.6% per year, with 40–70% of these patients having gallstones [2–4]. In China, acute biliary pancreatitis (ABP) is the most common form of pancreatitis [5]. The definitive treatment for ABP is cholecystectomy [6]. Currently, early cholecystectomy (EC) for mild acute biliary pancreatitis (MABP) has received a broader consensus and is also recommended by guidelines in several countries [6–10]. There is a general consensus in national guidelines that mild biliary pancreatitis is one of the surgical indications for cholecystectomy [1, 3–12]. In contrast, the contraindications to surgery for mild biliary pancreatitis are similar to those for laparoscopic cholecystectomy, such as American Society of Anesthesiologists (ASA) III patients > 75 years old; ASA IV and V patients; or pregnancy [5–13].

ABP requires attention not only to the surgical management of biliary disease, but also to the medical treatment of pancreatitis at the same time. AP can be divided into early and late stages. The early stage refers to the period from the onset to 2 weeks, and it is characterized by systemic inflammatory response syndrome (SIRS) and organ dysfunction [14, 15]. Controlling or reducing the inflammatory response in the early stage of AP can help in the recovery of patients. Laparoscopic cholecystectomy (LC) is the most common procedure in general surgery. However, there are still various factors that make it difficult to perform cholecystectomy, such as inflammation leading to peribiliary fibrous adhesions, peribiliary tissue, Calot’s triangle, and gallbladder bed edema [16]. There are anatomical difficulties in EC for pancreatitis [7]. In MABP, the incidence of difficult LC by a senior attending surgeon could reach 26% (29/112) [17].

Mirabilite, also known as Natrii sulfas or Glauber’s salt, is a hydrous sodium sulphate mineral with the chemical formula Na2SO4.10H2O. An artificial intelligence (AI) analysis showed that mirabilite is one of the four most commonly used herbs in the treatment of pancreatitis [18]. External application of mirabilite on the abdomen can reduce the exudation around the pancreas and the incidence of intra-abdominal hypertension (IAP) in severe acute pancreatitis (SAP) patients [19–21]. External use of mirabilite improves postoperative gastrointestinal mobility among older patients undergoing abdominal surgery [22]. External use of mirabilite reduces the incidence of pancreatitis in children after endoscopic retrograde cholangiopancreatographies(ERCP).It significantly alleviated post-procedural pain and reduced inflammatory response [23]. To date, there is no uniformity regarding the exact timing of EC for MABP [6]. Cholecystectomy within 72 h, 7 days, or 2 weeks can be defined as EC [6, 7, 24]. Moreover, patients undergoing surgery after symptom relief was defined as EC [6]. A treatment plan that promotes recovery from pancreatitis and reduces edema at the surgical site may help the patients undergo surgery more quickly and even shorten the hospitalization time. Based on previous studies, the aim of this study was to investigate the effect of external application of mirabilite on the inflammatory response of MABP and changes in the indexes associated with it.

## Materials and methods

### Participants

**Fig. 1:**
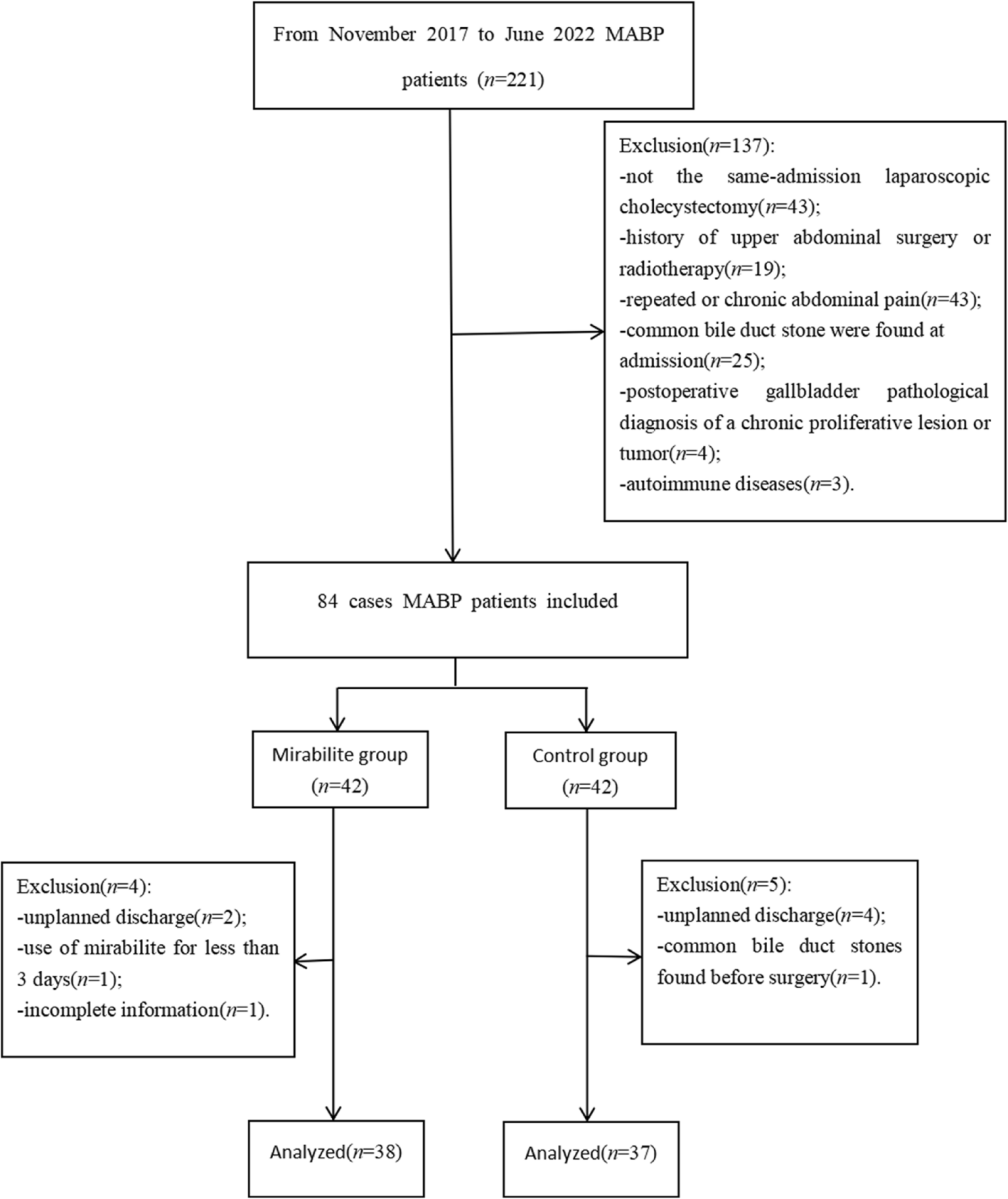
The filtering process: From November 2017 to June 2022 patients with mild acute biliary pancreatitis (MABP) (*n*=221). Exclusion (*n*=137) : not the same-admission laparoscopic cholecystectomy(*n*=43); history of upper abdominal surgery or radiotherapy (*n*=19) ; repeated or chronic abdominal pain (*n*=43) ; common bile duct stone were found on admission (*n*=25) ; postoperative gallbladder pathological diagnosis of a chronic proliferative lesion or tumor (*n*=4) ; autoimmune diseases (*n*=3) . The mirabilite group and the control group both had 42 cases patients, respectively. The mirabilite group: exclusion (*n*=4) : unplanned discharge (*n*=2) ; use of mirabilite for less than 3 days (*n*=1) ; incomplete information (*n*=1) . The control group: exclusion (*n*=5) :unplanned discharge (*n*=4) ; common bile duct stones found before surgery (*n*=1)

From November 2017 to June 2022, the medical records of MABP patients who underwent LC at the same time of hospitalization were analyzed. A total of 75 patients were included in the analysis: on the basis of the same treatment, the addition of mirabilite comprised the study group (38 cases) and the non-use of mirabilite comprised the control group (37 cases). Ultrasound and magnetic resonance cholangiopancreatograpy (MRCP) of the upper abdomen were completed in all patients within 24 h of admission. The diagnosis of acute pancreatitis is based on the fulfilment of two of three criteria: (1) upper abdominal pain, (2) serum amylase or lipase (or both) of at least three times the upper limit of normal, or (3) findings consistent with acute pancreatitis on imaging (contrast-enhanced CT, MRI, or abdominal ultrasound). Mild acute pancreatitis according to the Atlanta criteria: no local or systemic complications and organ failure [1]. Confirmed biliary etiology of the pancreatitis defined as: presence of gallstones or sludge on imaging, presence of common bile duct dilatation or elevation of the alanine aminotransferase two times higher than normal value [7]. Inclusion criteria were as follows: (1) Consistent with the diagnosis of MABP; (2) Cholecystectomy during the same hospitalization. Exclusion criteria were as follows: (1) Combined common bile duct stones; (2) Repeated or chronic abdominal pain; (3) History of upper abdominal radiotherapy or surgery; (4) Postoperative gallbladder pathological diagnosis of a chronic proliferative lesion or tumor; (5) Use of mirabilite for less than 3 days; (6) Autoimmune diseases; (7) Unplanned discharge; (8) Incomplete information. Each patient provided full informed consent. Patients and their families signed the consent form. This study was approved by the Ethics Committee of the Suining City Hospital of Traditional Chinese Medicine (No.2,022,040). The filtering process is shown in Fig. 1.

### Sample size calculation

This study was a controlled trial based on repeated measures ANOVA, with the study group being the addition of mirabilite group and the control group being the non-use of mirabilite group. The sample size was calculated based on whether there was a statistically significant difference in the time difference in the improvement of inflammatory indexes (blood cell count, C-reactive protein) between the two groups. Based on the pretest results, the sample size was calculated by applying online sample calculation software (http://powerandsamplesize.com*).* According to a certainty of 80% and α = 0.05, a sample size of 25 cases each was required for the study and control groups, and no less than 30 cases from each group were included in the analysis according to a 20% elution rate.

## Methods

The base treatment regimen was the same in both groups. In the study group, external application of mirabilite was initiated. In brief, 1000 g of mirabilite in a homemade bag was applied externally to the abdomen twice a day until surgery. Mirabilite was crushed into powder and placed in a double cloth bag (length about 30 cm and width about 20 cm). The bag was placed on the abdomen above the umbilical level, spread evenly, and properly secured with a lap band. Leakage of mirabilite was avoided to minimize skin irritation or damage. Hardened and slabbed mirabilite was removed.

LC was scheduled when the patient’s clinical symptoms disappeared and inflammatory indicators returned to normal. Ultrasound of the upper abdomen was performed in all patients within 24 h prior to surgery. The primary surgeon had experience of more than 100 cases of LC and was unaware of the patient’s preoperative treatment plan. LC were performed in all patients by the same surgeon using a “three-port approach.“ Gallbladder specimen was removed after being placed in a pick-up bag. Depending on the specifics of the procedure, the primary surgeon decided whether or not to place a drainage tube.

### Data collection and measurement

There were no statistical differences in any of the baseline data comparing the study and the control group (Table 1). The main indicators collected included the following: (1) inflammatory indicators: white blood cell count (WBC) and C-reactive protein (CRP) values on the 1st, 3rd, and 5th day of admission. (2) Symptomatic indicators: anal exhaust on the 1st, 2nd, and 3rd day after treatment. (3) Indicators related to surgery: gallbladder wall thickness (measured by ultrasound), adhesions score at the operative site, surgical drain placement, and operation time. A modified American Fertility Society (mAFS) score was used to assess adhesions at the surgical site: 0 indicated no adhesions (tenacity none, extent none); 1 indicated mild adhesions (tenacity filmy, extent < 25%); 2 indicated mild adhesions (tenacity filmy, extent 25–50%); 3 indicated moderate adhesions (tenacity filmy, extent ≥ 50%); 4 indicated moderate adhesions (tenacity dense, extent < 25%); 5 indicated severe adhesions (tenacity dense, extent 25–50%); and 6 indicated severe adhesions (tenacity dense, extent ≥ 50%) [25]. (4) Time indicators: waiting time for surgery and hospitalization time. In addition to the above indicators, we analyzed the occurrence of related adverse events, such as biliary fistula, bleeding, conversion to laparotomy, surgical site infection, and hospitalization due to the occurrence of biliary tract events within 3 months of surgery.

**Table 1:** Baseline characteristics of patients

Characteristics	Mirabilite group (*n* = 38)	Control group (*n* = 37)	*P* value
Male, *N*(%)	15(39.47%)	16(40.54%)	0.740^a^
Age in years, *M*(*SD*)	45.24(11.51)	48.38(10.75)	0.226^b^
BMI, kg/m^2^, *M*(*SD*)	22.25(1.60)	21.99(1.57)	0.474^b^
Hypertension, *N*(%)	9(23.68%)	10(27.03%)	0.739^a^
Diabetes, *N*(%)	6(18.75%)	7(18.92%)	0.720^a^
Smoking, *N*(%)	5(13.16%)	3(8.11%)	0.711^a^
Alcohol drinking, *N*(%)	7(18.42%)	5(13.51%)	0.562^a^
AMY, U/L, *M*(*SD*)	312.00(104.00)	325.76(117.48)	0.593^b^
LPS, U/L, *M*(*SD*)	226.26(73.44)	222.08(63.57)	0.793^b^
ALT, U/L, median (IQR)	125,50(101.50,150.50)	121.00(94.00,144.00)	0.626^c^
AST, U/L, median (IQR)	105.50(84.5,120.25)	98.00(81.00,117.50)	0.711^c^
GGT, U/L, *M*(*SD*)	142.21(47.52)	125.35(30.00)	0.070^b^
TBIL, U/L, *M*(*SD*)	45.63(12.31)	42.07(9.84)	0.172^b^
ALB, g/L	37.20(36.05,41.00)	37.80(35.80,40.60)	0.699^c^
Duration of symptoms before admission, hours, *M*(*SD*)	5.41(2.28)	5.35(1.96)	0.909^b^

*BMI *Body mass index, *HR(S) *Hours, *AMY *Blood amylase, *LPS *Blood lipase, *ALT *Alanine aminotransferase, *AST *Glutamic-oxalacetic transaminase, *GGT *Serum Gamma glutamyltransferase, *TBIL *Serum total bilirubin, *ALB *Serum albumin

^a^Used the chi-squared test

^b^Used the Independent-samples *T* test

^c^Used the Mann-Whitney U test

### Statistical analysis

Statistical analyses were performed using SPSS 25.0 (SPSS Inc.). Data were expressed as mean and standard deviation (*SD*) or proportion, and non-normally distributed variables were analyzed using medians and interquartile ranges. Continuous variables were compared by Student’s *t*-test or Mann–Whitney *U* test, and categorical variables were compared by chi-squared test or Fisher’s exact test, as appropriate. The Pearson correlation test was used to evaluate the correlation. Repeated measurement data were analyzed by repeated measures ANOVA. *P* < 0.05 was considered statistically significant.

## Results

### Inflammatory and symptomatic indicators

In the intra-group comparison, the WBC and CRP levels were reduced on the 1st, 3rd, and 5th days in both groups, and the differences were statistically significant (*P* < 0.01). In the mirabilite group, compared with the control group, the WBC on the 3rd day of admission and the WBC and CRP levels on the 5th day of admission decreased more rapidly and significantly by repeated measures ANOVA (*P* < 0.01). The descending speed in the mirabilite group was faster than that in the non-mirabilite group (Table 2). The mirabilite group had earlier anal exhaust time than the non-mirabilite group. Anal exhaust occurred mainly 2 days after treatment (Table 3).

**Table 2:** Preoperative white blood cell and CRP values

Group	Hospitalization time	WBC 10^9/L, *M*(*SD*)	CRP mg/L, *M*(*SD*)
**Mirabilite group(** ***n*** **= 38)**	The 1st day	14.36(1.19)	46.45(13.71)
	The 3rd day	12.04(1.18)^2) 5)^	26.66(7.33)^2)^
	The 5th day	8.89(1.25)^2) 4) 6)^	12.26(3.61)^2) 4) 6)^
**Control group(** ***n*** **= 37)**	The 1st day	14.50(0.86)	44.97(8.48)
	The 3rd day	12.57(0.79)^2)^	29.49(5.83)^2)^
	The 5th day	10.53(0.90)^2) 4)^	16.38(4.28)^2) 4)^

Comparison with the 1st day of hospitalization in the same group: ^1)^ *P<*0.05, ^2)^ *P<*0.01;

Comparison with the 3st day of hospitalization in the same group: ^3)^ *P<*0.05, ^4)^ *P<*0.01;

Comparison with control group at the same time point :^5)^ *P<*0.05, ^6)^ *P<*0.01.

All above data were analyzed by repeated measures ANOVA.

**Table 3:** Anal exhaust after treatment

	Mirabilite group (*n* = 38)	Control group (*n* = 37)	*P* value
Anal exhaust			
Day 1^a^	13(34.21%)	5(13.51%)	0.036^d^
Day 2^b^	33(86.84%)	20(54.05%)	0.002^d^
Day 3^c^	38(100.00%)	36(97.30%)	0.493^e^

^a^Number of anal exhaust patients after 1-day treatment

^b^Accumulative number of anal exhaust patients after 2-day treatment

^c^Accumulative number of anal exhaust patients after 3-day treatment

^d^Used the chi-squared test

^e^Used the Fisher’s exact test

### Surgery-related indicators

After treatment, the percentage of gallbladder wall thickness (≥ 3 mm) in the mirabilite group decreased from 71.05% (27/38, within 24 h of admission) to 42.11% (16/38, within 24 h before surgery), while the percentage of gallbladder wall thickness (≥ 3 mm) in the control group decreased from 72.97% (27/37) to 64.86% (24/37). The changes in gallbladder wall thickness were more pronounced in the mirabilite group than in the control group (*P* = 0.048). Mirabilite could relieve or stop thickening of the gallbladder wall. Compared with the control group, the mirabilite group had a low adhesion mAFS score (1.08 ± 0.59 points vs. 1.92 ± 0.60 points), few cases with drain placement (2/38 vs. 9/37), and a low operative time (38.03 ± 5.90 min vs. 48.51 ± 8.37 min), with statistically significant differences (*P* < 0.05) (Table 4). Analysis showed that both adhesion degree and gallbladder wall thickness (before surgery within 24 h) had a correlation with the operation time (*p* = 0.000, *r* = 0.821; *p* = 0.000, *r* = 0.458), respectively (Tables 5 and 6 ).

**Table 4:** Indicators related surgery

	Mirabilite group (*n* = 38)	Control group (*n* = 37)	*P* value
Gallbladder wall thickness(≥ 3 mm)			
Within 24 h of admission, n(%)	27(71.05%)	27(72.97%)	0.853^a^
Within 24 h before surgery, n(%)	16(42.11%)	24(64.86%)	0.048^a^
Adhesions mAFS score, points, *M*(*SD* **)**	1.08(0.59)	1.92(0.60)	0.000^b^
Surgical drain placement	2(5.26%)	9(24.32%)	0.020^a^
Operation time, minutes, *M*(*SD*)	38.03(5.90)	48.51(8.37)	0.000^b^

*mAFS *modified American Fertility Society

^a^Used the chi-squared test

^b^Used the Independent-samples *T* test

**Table 5:** The pearson correlation test shows the relationship between operation time and adhesions mAFS score

Correlations			
		Operation time (minutes)	Adhesions mAFS score(points)
**Operation time**	**Pearson correlation**	1	0.821^a^
	**Sig. (2-tailed)**		0.000
	**N**	75	75
**Adhesions mAFS score**	**Pearson correlation**	0.821^a^	1
	**Sig. (2-tailed)**	0.000	
	**N**	75	75

^a^Correlation is significant at the 0.05 level (2-tailed).

**Table 6:** The pearson correlation test shows the relationship between operation time and gallbladder wall thickness within 24 h before surgery

Correlations			
		Operation time (minutes)	Gallbladder wall thickness within 24 h before surgery(mm)
**Operation time**	**Pearson correlation**	1	0.458^a^
	**Sig. (2-tailed)**		0.000
	**N**	75	75
**Gallbladder wall thickness within 24 h before surgery**	**Pearson correlation**	0.458^a^	1
	**Sig. (2-tailed)**	0.000	
	**N**	75	75

^a^Correlation is significant at the 0.05 level (2-tailed).

### Outcomes of treatment

LC for MABP during the same hospital stay was safe and effective. There were no serious adverse events, such as biliary fistula, bleeding, surgical site infection, conversion to laparotomy, and hospitalization due to biliary tract events within 3 months after surgery, in both groups. Compared with the control group, the mirabilite group had a short waiting time for surgery (5.68 ± 0.70 days vs. 6.54 ± 0.59 days) and hospitalization time (8.95 ± 0.96 days vs. 9.84 ± 1.07 days), *P* < 0.05 (Table 7).

**Table 7:** Waiting for operation time and hospitalization time

	Mirabilite group (*n* = 38)	Control group (*n* = 37)	*P* value
Waiting time for surgery, days, *M*(*SD*)	5.68(0.70)	6.54(0.69)	0.000^a^
Hospitalization time, days, *M*(*SD*)	8.95(0.96)	9.84(1.07)	0.000^a^

^a^Used the Independent-samples *T* test

## Discussion

AP is an inflammatory condition of the pancreas that can cause local injury, systemic inflammatory response syndrome, and organ failure [15]. Biliary pancreatitis is the most common type of pancreatitis. The severity of biliary pancreatitis is classified according to the severity of the pancreatitis [6]. Biliary pancreatitis can be treated through medication and surgical intervention. In the past, studies on biliary pancreatitis have focused on the timing of surgery [6–10]. Although studies have shown that WBC and CRP can be used as predictors of the severity of biliary pancreatitis [26], there are limited reports on the recovery of inflammatory indicators during the treatment of biliary pancreatitis. The present study is the first to report that the external application of mirabilite can reduce the inflammatory response in MABP patients and shorten the time required for inflammatory indicators WBC and CRP to return to normal. Mirabilite can accelerate anal exhaust. The above effects of application of mirabilite accelerated the control of inflammatory response and disappearance of symptoms in MABP patients. These changes might help to shorten the waiting time for surgery and duration of hospitalization. These findings are similar to the results of previous studies, in which external application of mirabilite reduced the exudation, decreased the incidence of IAP, and improved gastrointestinal function in patients with severe pancreatitis [19, 21, 22].

The clinical efficacy of mirabilite is closely related to its biological mechanism. The 2020 edition of the *Chinese Pharmacopoeia* describes the main functions of mirabilite as follows: relaxing bowels, moistening dryness, softening hard stools, clearing fire, and reducing swelling. It is used for the treatment of real heat and stagnation, abdominal fullness and distension, dry stools, intestinal carbuncle, and swelling-related pain. It is also used for the external treatment of breast abscesses and painful swollen hemorrhoids [27]. Mirabilite can be taken orally as well as applied topically. External use of mirabilite can significantly reduce inflammatory cell infiltration, fibrous tissue hyperplasia, and thrombosis of vascular wall in rabbit ear vein. Mirabilite can significantly reduce the expression levels of interleukin (IL)-1, IL-6, and tumor necrosis factor-α (TNF-α) in mechanical phlebitis caused by a venous indwelling needle, and reduce the inflammatory response in rabbit ear vein [28]. The osmotic pressure produced by mirabilite is significantly higher than that in human tissues. When mirabilite is applied externally, water in the tissues is absorbed, thereby reducing the edema and improving local circulation. Mirabilite accelerates local lymphatic circulation, increases the phagocytosis of reticuloendothelial cells, and promotes the absorption of inflammatory cytokines [22].

The physiological mechanisms of mirabilite described above can help to explain our findings. We observed that the external application of mirabilite thinned the gallbladder wall. Gallbladder wall thickening is associated with inflammatory edema. Increased thickness of the gallbladder wall is a sign of significant changes in inflammation [29]. When the thickness of the gallbladder wall is ≥ 3 mm, there is a significant increase in intraoperative events during cholecystectomy, such as surgical drain placement, conversion to open surgery, bile spillage, bleeding, and bile duct injury. Gallbladder wall thickness may serve as an objective marker of LC complexity [29, 30].

In our study, it was found that more drains were placed in the control group than in the study group, 9/37 (24.32%) vs. 2/38 (5.26%), *P* < 0.05. There was no significant effect of whether drains were placed on intra-abdominal fluid, wound infection, post nausea vomiting, total length of hospital stay and postoperative death after laparoscopic cholecystectomy [31]. Although the placement of some of the drains was related to the operator’s habits, it was mainly based on intraoperative bleeding, biliary leakage/biliary fistula, organ damage and difficult operation [32]. The main reasons for the placement of abdominal drains in this study were analyzed to be related to the long operative time, significant adhesions in the operative area, and high intraoperative exudate. This is consistent with previous studies [30].

We also observed that the gallbladder in the mirabilite group had a much lower score of adhesions to the periphery and was dominated by sparse membranous adhesion. The formation of intra-abdominal adhesion may be related to the inflammatory response, coagulation, and fibrin deposition [33]. Continuous external application of mirabilite reduces the inflammatory response at the surgical site, thus promoting the decrease in edema of the gallbladder wall and interfering with the formation of adhesions. When the gallbladder wall is thinner and the adhesions are lighter, the operative time is likely to be shorter [20, 34, 35]. Our study showed that the operative times in the mirabilite group and the control group were 38.03 ± 5.90 (minutes) vs. 48.51 ± 8.37 (minutes), respectively, *P* < 0.01.

A systematic review studies on early cholecystectomy for MABP reported a conversion rate to open surgery of 7.27% (59/812) in LC [36]. This is inconsistent with our finding. The study suggests that the occurrence of surgical modality shift during laparoscopic cholecystectomy is associated with high CRP values, gallbladder gangrene, adhesions, unclear anatomy and history of previous abdominal surgery [36–38]. Our study excluded cases with previous abdominal surgery and recurrent chronic inflammation that could lead to peribiliary adhesions. In addition, with the popularity of laparoscopic surgery, techniques such as laparoscopic biliary exploration and laparoscopic bile-intestinal anastomosis are routinely performed in our center, and the rate of laparoscopic intermediate open surgery is lower than before. Advanced laparoscopic fellowship training decreases conversion rates during laparoscopic cholecystectomy for acute biliary diseases [39]. The lead surgeons involved in this study have extensive surgical experience.

There are previous scoring systems such as Nassar, Miyazaki and Parkland to respond to the degree of difficulty in cholecystectomy. However, Nassar, Miyazaki and Parkland grading systems primarily respond to the difficulty of surgical operation in laparoscopic cholecystectomy by means of a combined multiparameter score [16, 40]. The main effect of mirabilite is to reduce the inflammatory response in the operative area, and there is a close relationship between adhesions and inflammatory response. Since the mAFS adhesion score is only used for the evaluation of the degree of adhesions, it is more appropriate [25].

Cholecystectomy performed at 7 days after the resolution of MABP is an optimal timing that achieves a low incidence of recurrent biliary events before surgery as well as a low incidence of persistent choledocholithiasis and associated need for ERCP [7]. We also observed that LC was safe and effective in MABP patients with controlled symptoms at about one week after admission. Meanwhile, none of the patients experienced biliary fistulas, bleeding, conversion to laparotomy, surgical site infection, and the occurrence of biliary events at 3 months postoperatively.

However, this study has some limitations. First, due to the retrospective analysis, it was not possible to design different doses and frequencies of mirabilite treatment groups. The optimal dose and timing of mirabilite intervention could not be evaluated. Second, if more inflammatory indicators and gallbladder pathological parameters are available, it will help to explain the mechanism by which mirabilite reduces gallbladder wall edema and adhesion more comprehensively and deeply. Finally, single-center, small sample sizes are inadequate to assess adverse events. In the future, multicenter, prospective, randomized controlled studies could overcome the above-described shortcomings.

## Conclusion

The external application of mirabilite can reduce the systemic and local inflammatory responses, improve clinical symptoms and accelerate recovery in MABP patients. It might shorten the operation time by reducing edema and adhesions at the surgical site. Because of its characteristics, such as its being inexpensive, and easy to obtain and operate, mirabilite is suitable for clinical application.

## Data Availability

The datasets are not publicly available due to privacy or ethical restrictions but are available from the corresponding author on reasonable request.

## Abbreviations

MABP: Mild acute biliary pancreatitis
LC: Laparoscopic cholecystectomy
WBC: White blood cell
CRP: C-reactive protein
mAFS: modified American Fertility Society
AP: Acute pancreatitis
EC: Early cholecystectomy
SIRS: Systemic inflammatory response syndrome
IL: Interleukin
